# Effect of Agricultural Biomass Residues on the Properties of Recycled Polypropylene/Polyethylene Composites

**DOI:** 10.3390/polym15122672

**Published:** 2023-06-14

**Authors:** Agnese Ābele, Remo Merijs-Meri, Madara Žiganova, Zanda Iesalniece, Ivan Bochkov

**Affiliations:** Faculty of Materials Science and Applied Chemistry, Institute of Polymer Materials, Riga Technical University, LV-1048 Riga, Latvia

**Keywords:** reinforcement, agricultural biomass waste, sweet clover straws, buckwheat straws, rapeseed straws, recycled polyolefin

## Abstract

The aim of the study was to assess the usefulness of agricultural biomass residues as reinforcement in recycled polymer matrices. In this study, recycled polypropylene and high-density polyethylene composites (rPPPE) filled with three types of biomass residues, sweet clover straws (SCS), buckwheat straws (BS) and rapeseed straws (RS), are presented. The effects of the fiber type and the fibers content on the rheological behavior, mechanical properties (including tensile, flexural and impact strength), thermal stability and moisture absorbance were determined, in addition to morphological analysis. It was revealed that the addition of SCS, BS or RS increased the materials’ stiffness and strength. The reinforcement effect increased as the loading of the fibers was increased, especially for BS composites in the flexural test. After the moisture absorbance test, it was found that the reinforcement effect slightly increased for the composites with 10% fibers but decreases with 40% fibers. The results highlight that the selected fibers are a feasible reinforcement for recycled polyolefin blend matrices.

## 1. Introduction

Currently, biobased composites are becoming more and more common. Increasing interest in recent decades is related to the circular economy paradigm involving the maximum use of resources from waste streams. Biobased polymer composites are recognized by their high stiffness, lightness, sustainability and resource-efficiency. Different natural fibers, for example, leaf fibers, bast fibers, reed fibers, seed fibers and wood fibers [1], are used as reinforcing fillers for biobased polymer composites. For example, because of their superior mechanical properties, hemp fibers are extensively used in polymer composites [2]. Selection of fibers depends also on local availability of industrial and agricultural process residues, for example, grain processing residues.

This current research assessed the use potential of local biomass waste left in the field, such as sweet clover (SCS), buckwheat (BS) and rapeseed straws (RS), for development of composites based on recycled polymers. According to the scientific literature, selected straws have not extensively researched as fillers in polymer composites. Rigal M. et al. characterized two sweet clover species and evaluated their possible use as a source of fibers. In the result, they concluded that sweet clover fibers have potential as a natural reinforcement for use in polymer composites [3]; however, to the authors knowledge, there are no more studies on the application of SCS in polymer composites. Buckwheat straw reinforcement in polymer compositions has been investigated slightly more. Andrzejewski J. et al. evaluated the effect of buckwheat husk fibers on different matrices, such as isotactic polypropylene, linear low-density polyethylene, and poly (lactid acid) biopolymer, suggesting that they can be used to replace the widely used wood fibers [4,5]. From the selected three biomass types, rapeseed straws have been investigated the most. Several studies have been performed by Paukszta D. et al. [6,7,8]; they evaluated a mercerization process for rapeseed straw by investigating the influence of rapeseed straw reinforcement on the polypropylene matrix crystallization process [7]. They also compared the influence of healthy rapeseed fibers and those infested with the *S. sclerotiorum* fungus on a polypropylene matrix’s mechanical properties [8]. Bichi A.H. et al. [9] studied the influence of treated and untreated fibers on the structural, thermal and mechanical properties of polyvinyl chloride matrix composites. Furthermore, rapeseed fibers were also assessed as a replacement for wood particles in particleboard production [10,11].

By considering the growing levels of post-consumer polymeric waste, a commercial mix of recycled polypropylene (PP) and recycled polyethylene (PE) was used in this research. PP and PE both are widely used in packaging and in the warehouse, consumer and household, building and construction, automotive and other sectors [12]. The global demand for PP and PE is expected to increase by approximately 38 million metric tons in 2023 compared with 2017 (about 180 million tons in total) [13]. The both polymers in a post-consumer waste stream are often mixed together, forming a mechanical mix (rPPPE) and there are technical difficulties and economic drawbacks to their separation in the recycling process due to structural similarities and close density values. Aumnate C. et al. [14] reported on the influence of low and high density polyethylene on PP crystallization behavior and tensile properties. By considering the thermodynamic incompatibility between PP and PE, they observed that the lack of interfacial adhesion was strongly correlated with the shape of the crystal and the crystallization rates. They concluded that the tensile properties of the composite depends on the composition and the polyethylene type, especially at higher polyethylene loads.

In the scientific literature, there is a number of investigations on recycled PP or recycled PE composites with natural fibers. For example, Srebrenkoska V. et al. [15] demonstrated that recycled PP with kenaf fibers and rice straw fibers shows mechanical and thermal behaviors similar to virgin PP composites. The authors recycled the virgin PP twice to compare the results of the PP and rPP. The module of elasticity increased up to 2300 MPa at 30% concentration of the kenaf fibers compared with PP matrix (1081 MPa). The flexural strength changes were insignificant in the kenaf composites’ case but for the composite with rice straws, a decrease from 49.5 to 39 MPa was observed. Increases in tensile strength, flexural strength and modules of elasticity were also observed in Abdullah M. Z. et al.’s study on recycled PP with 40% mengkuang leaf fibers [16]. At this fiber concentration, the tensile module of the composite increased by 224%, reaching 5.1 GPa, the flexural module increased from 0.93 to 2.23 GPa, and the tensile strength increased by 27.6% (from 18.1 to 23.1 MPa), but the flexural strength increased by 38% (from 25.3 to 34.9 MPa). Dorneles de Castro B. et al. [17] investigated recycled a PE matrix with sisal fibers and obtained composites with improved tensile strength and stiffness, changed from 13.72 to 18.29 MPa and increased module of elasticity from 730 to 1450 MPa. To ensure high-performance characteristics of recycled polymer matrix composites with reinforcing fibers from biomass, their compatibilization is usually required. In spite of some attempts to perform compatibilization of recycled polymer matrix composites with natural fibers by using renewable compatibilizers [18], they have not yet gained commercial success due to the high price, necessity to use labelled chemicals for its synthesis, and usually insufficient efficiency in comparison to such universal and commercially viable compatibilizers such as maleic anhydride groups containing grafted polyolefine [18,19]. This was the basis for the use of maleic anhydride-grafted polypropylene compatibilizer in this research.

In our previous works [20,21], by considering the fibers’ surface morphology and aspect ratio it was concluded that sweet clover, buckwheat and rapeseed straws’ biomass residues have a promising potential to improve the mechanical properties of recycled PPPE. The surface accessibility of the fibers from the given agricultural biomass residues was increased because of their increased purity after sodium hydroxide (NaOH) and N-methylmorpholine N-oxide (NMMO) treatment, which may promote mechanical interlocking and improve compatibility with the polymer matrix. In respect to aspect ratio as one of factors for successful reinforcement, it was demonstrated that aspect ratios of NaOH-treated RS, BS and SCS were 12, 9 and 5, respectively. The aim of this study was to investigate the influence of the pre-treated RS, BS and SCS on the mechanical, rheological and moisture resistance properties of the PPPE-based composites in a broad weight concentration range of the filler (0 to 40 wt.%).

## 2. Materials and Methods

### 2.1. Materials

**Figure 1:**
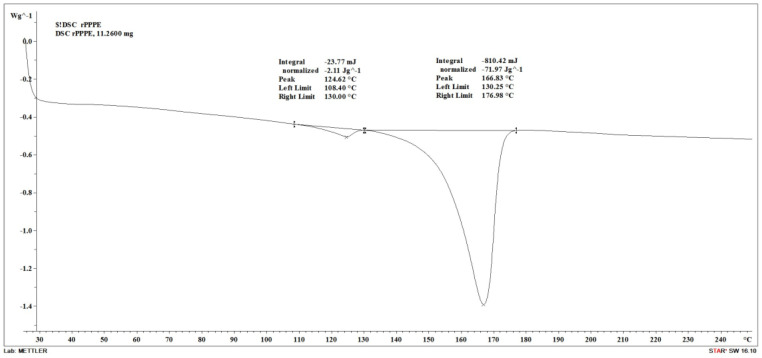
DSC thermogram of rPPPE as supplied.

Recycled polypropylene and polyethylene mix (rPPPE) produced from large bags was provided by Ltd. Nordic Plast (Olaine, Latvia). In Figure 1, the differential scanning calorimetry (DSC) thermogram of the polymer matrix is shown; it reveals two distinct melting peaks, one corresponding to high density polyethylene (at 125 °C) and the second to polypropylene (at 167 °C). According to the supplier, the rPPPE exhibited a melt index of 7 g/10 min. Maleic anhydride-grafted propylene (MAH) was obtained from Merck (Darmstadt, Germany).

A stabilizer, IrgaCycle UV033DD (IRGA), was used to prevent degradation of the material during thermomechanical processing. IRGA was donated by Ltd. BASF SE (Ludwigshafen, Germany).

Winter rapeseed straw (RS), buckwheat straw (BS) and sweet clover straw (SCS) were collected as biomass waste from the local farms Braslini, Pasiles, and Susuri. The fibers were grinded and mercerized before introduction into the rPPPE matrix [20].

### 2.2. Methods

#### 2.2.1. Composites and Test Sample Preparation

Compounding of the composites was carried out by using a two-roll mill from Lab Tech Engineering Company at the respective roll temperatures (167 °C and 172 °C) and rates (20 rpm and 28 rpm). The compounding process took 10 min, during which, the first polymer matrix was melted, then 3 wt.% of MAH and 1 wt.% of IRGA were added, and lastly, the fibers were gradually added. The amount of the added fibers was varied in a wide range (0–40 wt.%). Table 1 presents the developed composites and their compositions. After melt compounding, the composites were ground with a Retsch SM300 rotary grinder at a speed of 900 rpm using a 6 mm sieve; this was followed by injection molding (Minijector 55) to obtain the samples required for the testing of mechanical properties in quasistatic tension and bending, as well as impact loading tests. The temperature profile of the barrel during the injection molding was from 170 to 190 °C. The samples for the rheological tests were prepared by compression molding using a Carver CH 4386 hydraulic press at 190 °C and a pressure of 5 MPa. The dimensions of the sample were 25 mm in diameter and 1.2 mm in thickness.

#### 2.2.2. Analysis of Properties

##### Differential Scanning Calorimetry (DSC)

The DSC thermograms of the matrix were obtained using a differential scanning calorimeter DSC1/200 W (Mettler Toledo, Germany) from 25 °C to 250 °C at a rate of 10 °C/min in air flow. The degree of crystallization was calculated using Equation (1):

(1) $$\mathrm{Xc}\left(\%\right)=\frac{\Delta \mathrm{Hm}}{\Delta \mathrm{Ht}\;\cdot ɸ}\;\cdot 100$$

where Xc is the degree of crystallinity (%), ∆Hm is the enthalpy of melting of the sample (J/g), ∆Ht is the theoretical PP enthalpy of crystallization (209 J/g), and ɸ is the mass fraction of the PP in the composites.

##### Scanning Electron Microscopy (SEM)

The fracture morphologies of the composites were analyzed with a Tescan Mira/LMU scanning electron microscope operated at 5 kV and 5k×, 1k× and 300× magnification. The samples’ fracture surfaces after the Charpy impact tests at −160 °C were coated with a gold layer using an Emitech/Quorum K550X plasma-sputtering apparatus.

##### Tensile Properties

The tensile test was conducted according to LVS EN ISO 527-1:2020 and LVS EN ISO 527-2:2012. The tensile properties were measured at room temperature (+23 ± 2 °C) and humidity (50 ± 5%) using a Zwick/Roell BDO-FB020TN universal testing machine with pneumatic clamps, 2.5 kN pancake-type load cell of accuracy class No. 1 and mechanical contact extensometer Zwick/Roell B066550.BL of accuracy class No. 0.5. The preload was 10 N. The speed during the determination of Young’s modulus at a deformation interval of 0.05–0.25% was 1 mm/min, whereas after the modulus was determined, the speed was 50 mm/min until breaking of the test specimens.

##### Flexural Properties

The flexural test was performed according to LVS EN ISO 178:2019. The flexural properties were measured at room temperature (+23 ± 2 °C) and humidity (50 ± 5%) using a Zwick/Roell BDO-FB020TN universal testing machine with a 3-point flexural test stand and 2.5 kN pancake-type load cell of accuracy class No. 1. For the tests, a 10 N preload as well as a constant 2 mm/min testing speed until 16 mm flexure were used.

##### Impact Strength

The non-instrumented Charpy impact test was performed in accordance with LVS EN ISO 179-1:2010. The impact properties were measured at room temperature (+23 ± 2 °C) and humidity (50 ± 5%) using a Zwick/Roell 5102 impact machine with 4 J pendulum. Un-notched standard samples of type 1 were tested edgewise.

##### Rheological Properties

The rheological tests were performed using an Anton Paar Smart Pave 102 dynamic shear rheometer. The amplitude and frequency sweep tests were performed at 190 °C using a plate–plate system, with plate diameters of 25 mm and gap of 1 mm. The strain amplitude sweep tests were performed at a constant frequency of 1 rad/s and increasing shear strain. The shear strain for all the samples was between γ = 0.1–1000%. The frequency sweep tests were carried out in the frequency region between ω = 0.01–100 rad/s and at a constant shear strain of γ = 5%. The value of shear strain was selected in the linear viscoelastic (LVE) region of the material as determined by the strain amplitude sweep tests.

##### Moisture Absorption Test

The water absorption test was determined according to EN 326-1. The flexural samples were immersed in distilled water at 23 °C ± 2 °C for 2 weeks. The samples were weigheded before the test as well as after one and two weeks using a Precisa Instruments AG analytical balance with a precision of 0.001 mg. Water absorption was determined using Equation (2).

(2) $$\Delta W\left(\%\right)=\frac{\left(W2-W1\right)}{W1}\;\cdot 100\;$$

where ΔW is the water absorption (%), W1 is the initial weight (mg) and W2 is the final weight (mg).

##### Thermogravimetric Analysis (TGA)

The determination of thermal stability of the investigated composites was performed using a Mettler Toledo TGA/DSC 3+ thermogravimetric analyzer. The TGA was performed from 25 to 600 °C with a heating rate of 10 °C/min under an air atmosphere with a flow rate of 60 mL/min.

## 3. Results and Discussion

### 3.1. Mechanical Properties of the Composites

The mechanical performance of polymer composites is dependent on the matrix and fiber properties, as well as interfacial interactions. Unfortunately, the maximum reinforcement effect of biobased composites usually cannot be achieved due to intrinsic incompatibility between the hydrophilic fibers and hydrophobic matrix, the fibers’ low aspect ratio, and insufficient fiber concentration in the composite [1,2,22]. To ensure maximum reinforcement, the proper selection of the composite development method is also essential. In the current study, melt compounding by two roll mills was selected.

**Figure 2:**
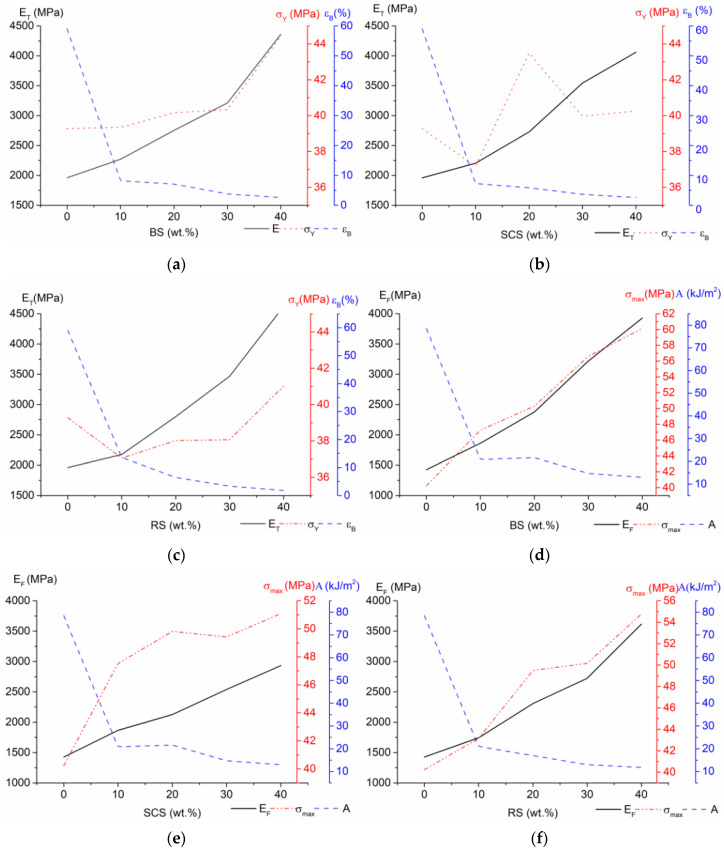
Tensile modulus (E_T_), tensile strength (σ_Y_) and strain at break (ε_B_) of rPPPE composites with (**a**) BS, (**b**) SCS and (**c**) RS fibers and flexural modulus (E_F_), maximum flexural stress (σ_max_) and impact strength (A) of rPPPE composites with (**d**) BS, (**e**) SCS and (**f**) RS fibers.

The tensile and flexural properties of the composites with BS, SCS and RS are presented in Figure 2, and Tables S1 and S2. It is known that the modulus of elasticity of polymer composites with lignocellulosic fillers typically increase with increasing fiber loading due to the rigid mechanical properties of the filler. However, it usually leads to a decrease in strain at break. As it can be seen, by increasing the lignocellulose fiber content, the stiffness of the investigated composites significantly increased; for an example, in the BS case, E_T_ increased by 16% (10% BS), 40% (20% BS), 64% (30% BS) and 122% (40% BS), but the strain decreased by 86% (10% BS), 88% (20% BS), 94% (30% BS) and 96% (40% BS). A similar tendency was observed from the flexural test data (see Figure 2d–f), where only the E_F_ increments were higher, i.e., E_F_ increased by 30% and 175% with the addition of 10% and 40% BS fibers, respectively. Comparing the influence of the fiber types used, it was observed that all three fibers provided a very similar E increase.

By further evaluating the reinforcement effect of the investigated fibers, one can see that under tensile load yield stress, the σ_y_ values changed slightly in the range of 2% for BS-, 5% for RS- and 10% for SCS-containing composites. The increase was more prominent in the case of the maximum flexural stress σ_max_; by increasing the fiber amount, the flexural strength gradually increased and reached its maximal value at 40% fiber content in the composite. At a 40% concentration, the BS-containing composite presented the highest σ_max_ value (60 MPa), equal to a 50% increase in comparison to neat polymer matrix. The SCS composites showed the lowest flexural strength with a maximum increase of 27%. However, given that SCS has the smallest aspect ratio (AR = 5) and the shortest fiber length (mostly 400 µm), the change was still significant. In spite of the fact that RS possessed the highest AR (12) and the longest fiber length (around 700 µm), the increase in σ_max_ was lower than that of the BS-containing composites.

One factor which affects mechanical properties is crystallinity. In the literature, two opposite trends may be found: the crystallinity degree (Xc) increases due to the fibers acting as nucleating agents [23] or the crystallinity degree does not change much with increasing fiber content [24]. In the case of the investigated composites, it was found that the fibers induced crystal nucleation of the polymer matrix. HDPE was apparently present in a small amount and changes in its melting enthalpy did not significantly affect the overall degree of crystallinity, unlike PP. The melting enthalpy of PP decreased with increasing fiber content. The degree of crystallinity of the PP fraction (calculated using Equation (2)) in the rPPPE was 36%. For the composites, Xc increased in a range from 36 to 47%. The maximum melting temperatures of the investigated composites did not change significantly (less than 3 °C).

As expected, by increasing stiffness, the impact strength of the investigated composites was reduced due to limited chain mobility caused by the addition of the fibers as well as the development of weak interfacial regions due to intrinsic incompatibility between the polymer matrix and the lignocellulosic filler. Weak interface regions, similarly to agglomerates, contribute to the propagation of cracks. Moreover, intense formation of cracks may be also attributed to insufficient fiber length and increased amount of fibers ends limiting efficient stress transfer from the matrix to the fibers [25].

### 3.2. Scanning Electron Microscopy (SEM)

**Figure 3:**
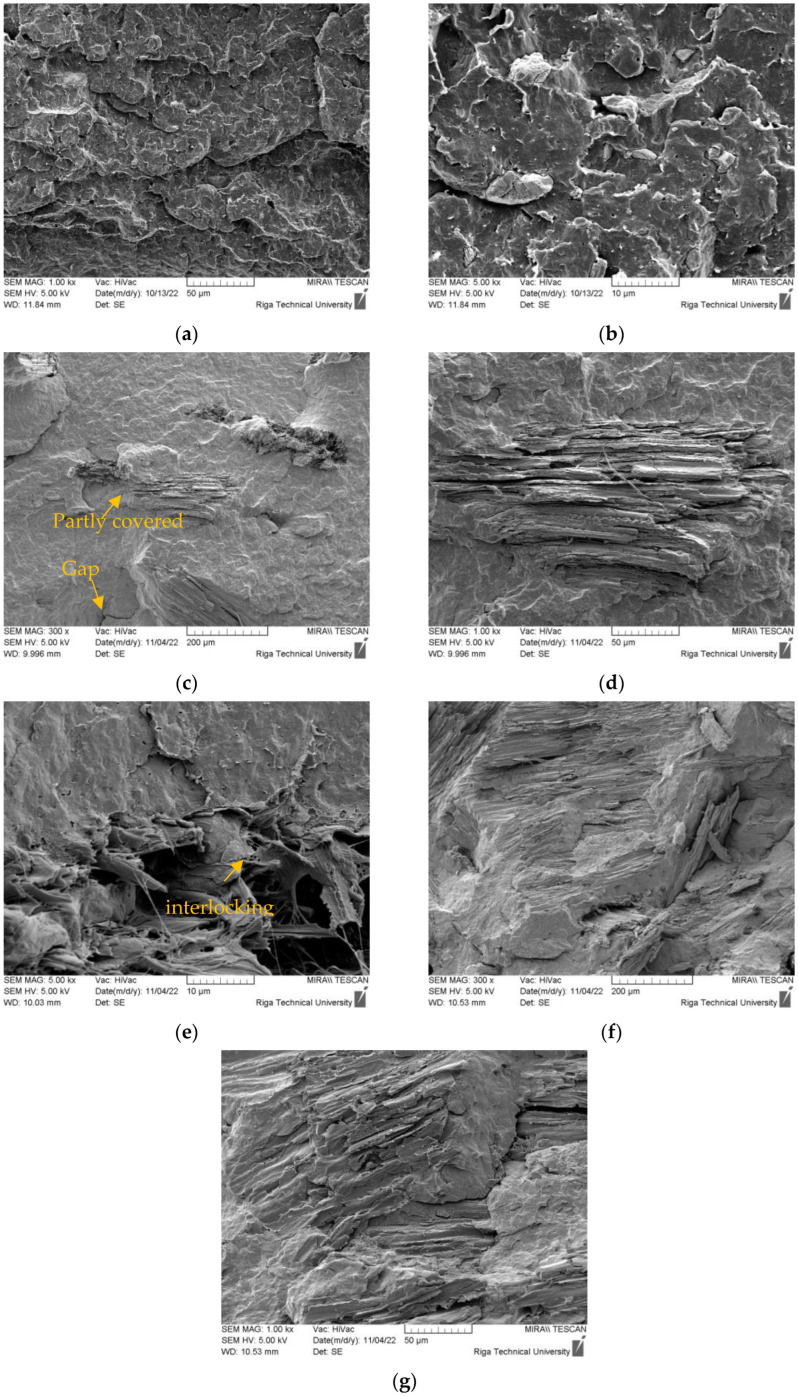
Scanning electron microscopy (SEM) images of (**a**,**b**) rPPPE at magnifications of 1k× and 5k×; as well as the composites with (**c**–**e**) 10% SCS and (**f**,**g**) 40% SCS at magnifications 5k×, 1k× and 300×.

SEM micrographs of the fractured surfaces of the rPPPE matrix and its composites with SCS are shown in Figure 3. The investigated composites with other lignocellulosic fibers possessed similar morphological features; therefore, SEM micrographs of only the SCS-reinforced composites are shown as an example. It can be seen (Figure 3a,b) that the polymer matrix consists of two polymers, and the minor polymer phase is not uniformly dispersed. The rPPPE was obtained from recycling of large bags, which are mainly made of polypropylene and a small percentage of polyethylene. Therefore, there are irregular particles of polyethylene, which are surrounded by polypropylene matrix. In general, the polymer matrix has a coarse morphology with small holes and gaps between the polymers, which accounts for the low interfacial adhesion. It is well established that by adding lignocellulose fibers into a polyolefin matrix, a lack of chemical interaction will be observed due to the considerably different polarities of the components. Therefore, the morphologies of the composites with 10% and 40% fibers reveal the presence of gaps between the matrix and the fibers, which are partly pulled out or broken. Nonetheless, as expected from research on the fiber pre-treatment [20,21], the matrix was mechanically interlocked between the fiber layers, i.e., the fibers were covered by the polymer matrix (see Figure 3e). In some cases, incomplete covering was observed, probably due to the specific fibers’ microstructure. In addition to surface roughness of the fibers, another important contributor for the formation of an interlayer between the phases is the use of maleic anhydride as the coupling agent; it contributed to the formation of the interlayer, which enabled stress transfer between the fibers and the matrix, resulting in improved mechanical properties [26]. It was also observed that the fiber orientation was random; this is another factor that influences the mechanical properties of reinforced composites. In general, the SEM results were in accordance with the results of the mechanical tests and the interaction between the components of the composite still can be improved. In the literature, there are several studies devoted to the optimization of the quantity of the compatibilizer and use of different approaches for fiber treatment in order to minimize the incompatibility (gaps and voids) between the fibers and polymer matrix [27,28].

### 3.3. Rheological Properties

**Figure 4:**
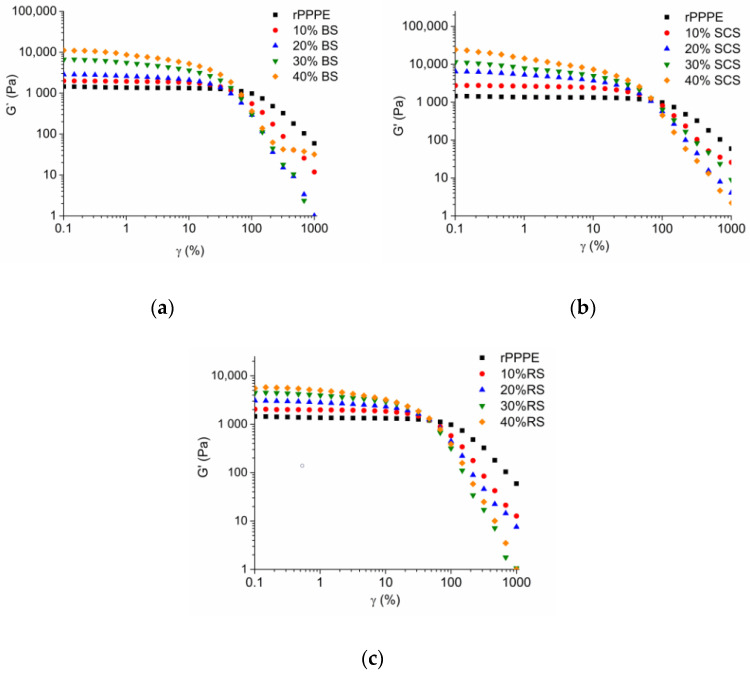
Storage modulus (G′) versus strain (γ) at 190 °C for rPPPE composites with (**a**) BS; (**b**) SCS; (**c**) RS.

Information about the interactions between the polymer and the fibers of the investigated composites can also be obtained from rheological measurements. From the strain sweep test (Figure 4), it can be seen that by increasing the fiber content, the storage modules of the composites gradually increased; at the maximum fiber content (40%), G′ almost reached 24,256 Pa, 8650 Pa and 5535 Pa for SCS-, BS- and RS-containing composites at a 0.1% strain. However, the linear viscoelasticity (LVE) region decreased with increasing fiber content in the composites. The G′ of the composites decreased faster than that of rPPPE, especially the BS composites. With 10% fiber, the composites’ rheological behaviors were similar to the matrix, but for the composites with higher fiber contents, the LVE behavior ended at smaller strains. According to Pušnik Črešnar K. et al. [29], the fibers form denser network in the composites with a higher amount of fibers; the resulting material is stiffer but shear disrupts this network quicker. As mentioned in the literature, rheological behaviors in the strain/stress and frequency tests depend on the interface; low compatibility between components does not provide effective absorption of the deformation energy [30].

**Figure 5:**
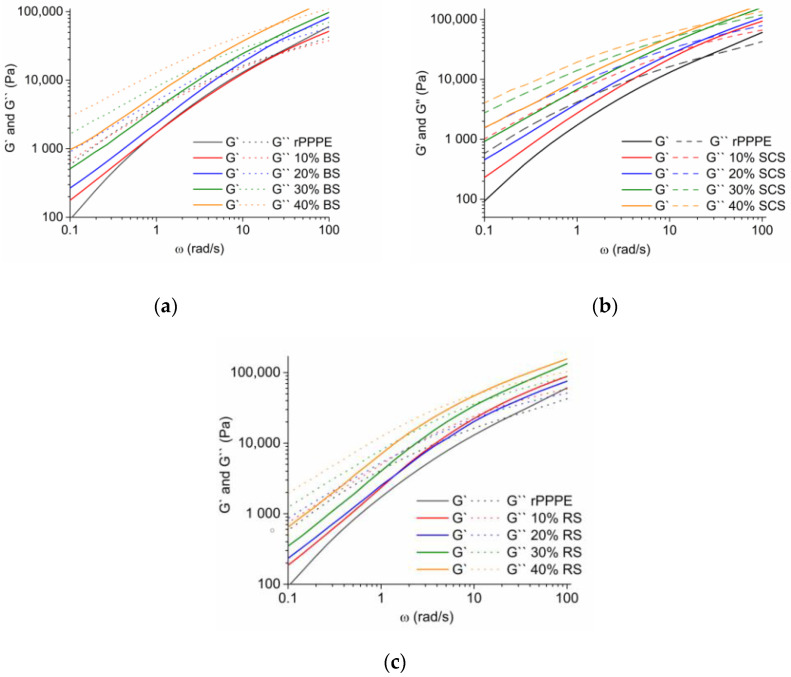
Storage modulus (G′ as solid lines) and loss modulus (G″as dashed lines) as a function of angular frequency at 190 °C for rPPPE composites with (**a**) BS, (**b**) SCS and (**c**) RS.

The lignocellulose fibers were more rigid than the polymer matrix, as reflected in the higher storage (G′) and loss (G″) modules obtained from the frequency tests of the composites. In Figure 5, one can see that, at low shear rates, all the composites had more a viscous behavior (G″ > G′) but at higher frequencies, elastic behaviors started to dominate. Furthermore, the G′ and G″ values of the composites were higher than those of rPPPE.

From Figure 5, the crossover point (Gc) was determined and is represented in Table 2. The data reveal that the Gc values significantly increases with the addition of the fibers, which is the typical behavior for filled systems.

**Figure 6:**
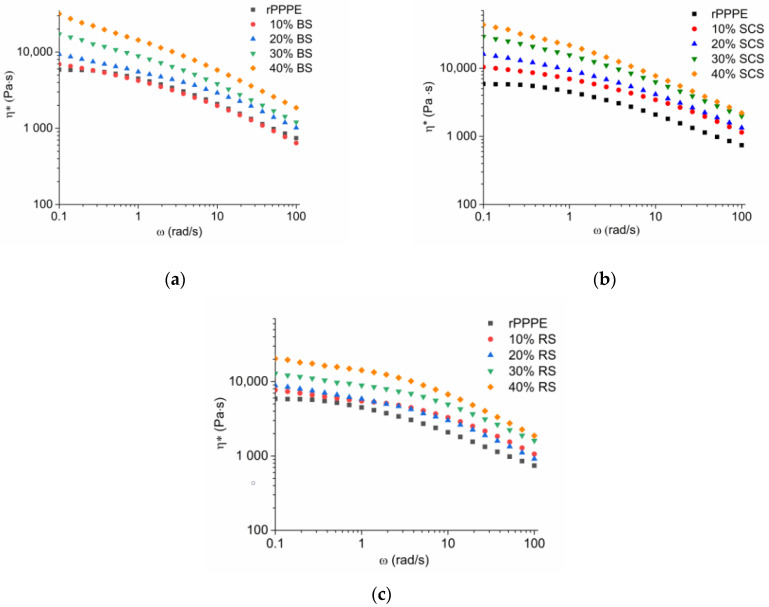
Complex viscosity (η*) as function of angular frequency at 190 °C for rPPPE composites with (**a**) BS; (**b**) SCS; (**c**) RS.

The complex viscosity (η*) of the investigated composites is depicted in Figure 6, from which it may be seen that the η* values increases with the addition of fibers into the polymer matrix because of the hindered mobility of chain segments in a melt flow. It was noticed that the viscosities of the composites increased gradually with increasing fiber concentration and reached the maximum values at the highest fiber concentration. For example, at 0.1 rad/s, the η* of the 10% composites was 6923 Pa·s, 10,367 Pa·s and 7696 Pa·s, for the BS-, SCS- and RS-containing systems, respectively. At the highest fiber loading (40%), the viscosity reached 31,613 Pa·s, 43,359 Pa·s and 20,371 Pa·s for the BS-, SCS- and RS- containing composites, respectively. The difference in rheological behavior between the three types of fibers may be caused by several factors such as the fiber’s size, shape, hardness, surface area, agglomeration, etc. In this case, fibers size is likely the main reason for the higher viscosity values. There is evidence of a strong correlation between viscosity and fiber size, consistent with the finding of other research teams [30,31]. The highest value of viscosity was shown by the composites containing SCS, which had the shortest fibers. Concomitantly, the lowest viscosity was observed for the composites containing longer RS fibers. For example, at 0.1 rad/s, the viscosity of the composite with 40% RS was 247% higher but 638% with 40% SCS fibers in comparison to rPPPE. In the BS composites, the maximal viscosity change reached was ~438%. By increasing frequency, the viscosity decreased due to the shear thinning effect, which is known to be typical for pseudoplastic materials such as thermoplastic polymers and their composites [30,32].

### 3.4. Moisture Absorption

**Figure 7:**
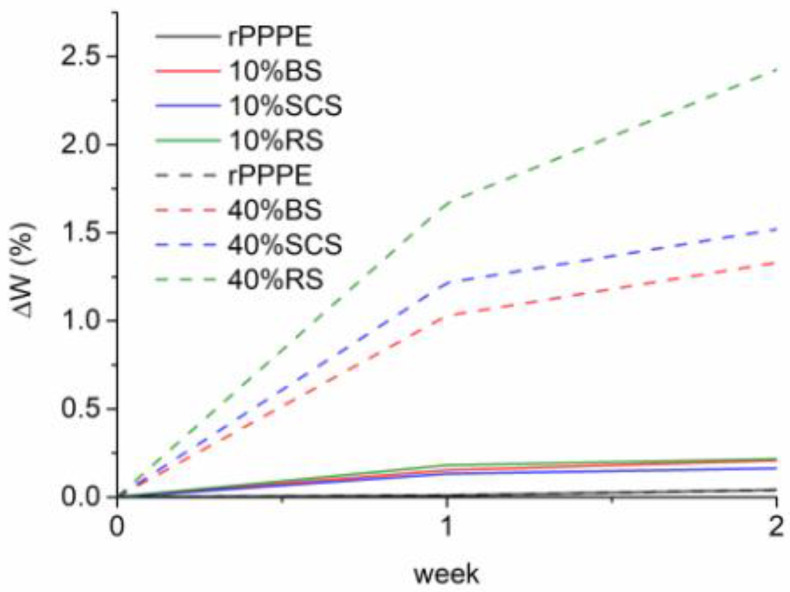
Moisture sorption curves of rPPPE and the composites with 10% and 40% lignocellulosic fibers (ΔW%).

Lignocellulosic fibers have a hydrophilic nature due to hydroxyl (OH) groups in the amorphous region of the fiber; the OH groups form hydrogen bonds with water molecules from the external environment [22,33]. This is an important factor to consider in the development of composites, in particular with non-polar polymers, because it may limit biobased composites’ use in environments where water can be accessed. After the fiber treatment, the surface impurities are removed and the surface becomes less polar and more hydrophobic [34]. In Figure 7, the moisture absorption kinetics of the investigated composites are shown. It was already expected that water absorption will increase by adding the lignocellulosic fibers. At higher fiber amounts, water absorption significantly increased, especially in the case of the RS-containing composites. After 2 weeks of water immersion, neat rPPPE demonstrated slight water absorption (W = 0.04%). Meanwhile, for the composite with 10% lignocellulosic fillers, ΔW increased up to 0.26, 0.21 and 0.22% for the SCS, BS and RS composites, respectively. At the highest fiber content (40%), ΔW was 1.52, 1.33 and 2.05%, respectively for the composites with SCS, BS and RS. These differences could be related to the chemical compositions of the fibers; for example, RS fibers are characterized by the highest hemicellulose content [20]. However, additional considerations need to be taken into account such as surface cleanness, composite adhesion and possible cracks.

**Figure 8:**
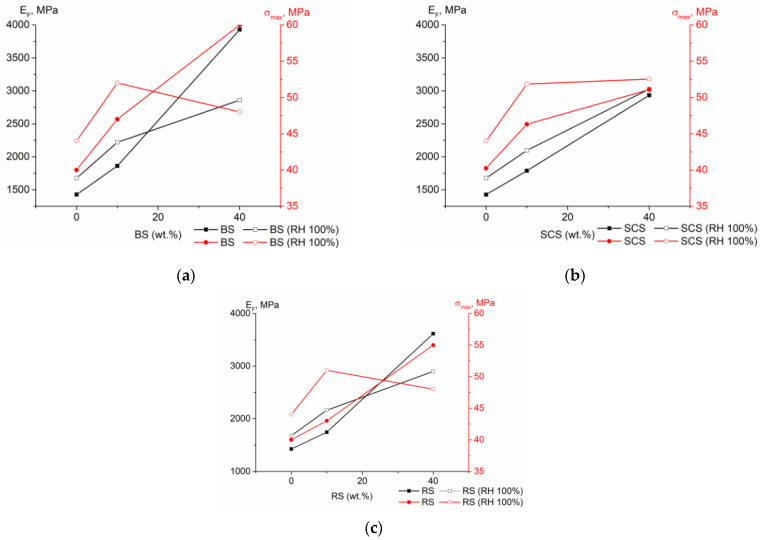
Flexural module (E_F_) and flexural strength at maximum (σ_max_) of the rPPPE composites with (**a**) BS, (**b**) SCS and (**c**) RS fibers. (Blank symbols relate to the composites after immersion test).

At the same time, changes in flexural properties were assessed after the immersion test. The flexural properties before and after immersion of the composites with BS, SCS and RS are presented in Figure 8 and Table S2. The obtained results indicate that the elastic modules of the investigated composites increased at a 10% fiber content. The highest increase (23.88%) was observed for the 10% RS composite while the modulus of the corresponding composites with BS and SCS increased by 10.63% and 11.95%, respectively. The flexural strength also increased, but only for the 10% composites; at a higher fiber loading, σ_max_ showed a decrease. At the same time, the deformation at maximum flexural stress values remained constant. The noted changes after immersion in water can be attributed to the swelling process of the fibers. It was observed that with small amount of fibers swelling had a positive effect. However, at a higher fiber loading in the composites, a decrease in strength was observed, probably due to the swelling caused by debonding of the fibers [33].

### 3.5. Thermal Properties

**Figure 9:**
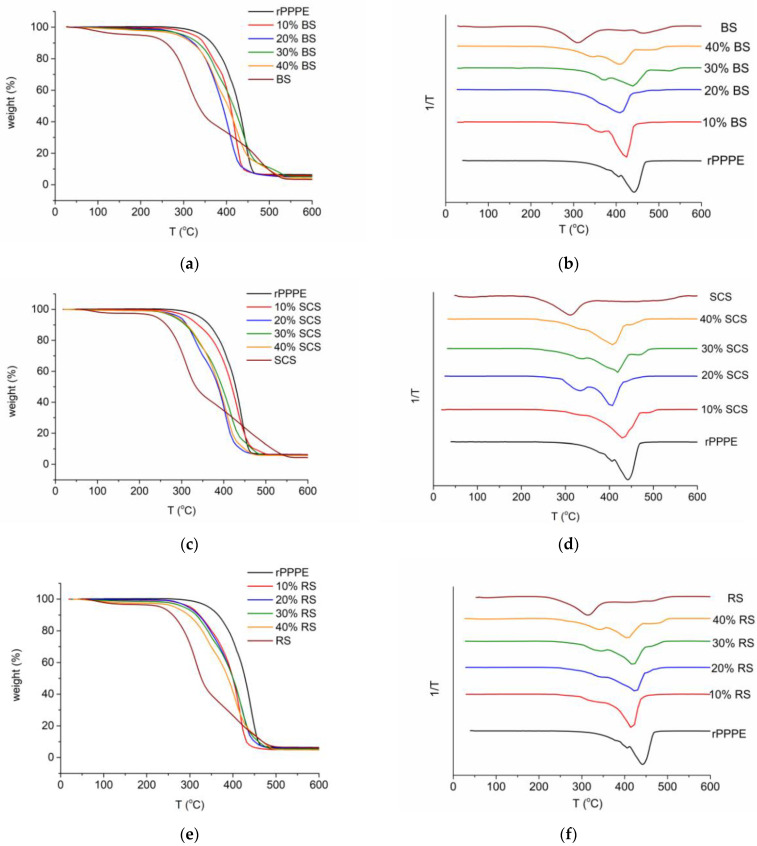
TGA (**a**,**c**,**e**) curves and DTG (**b**,**d**,**f**) curves of the investigated composites.

The thermal behaviors of the rPPPE and its composites with natural fibers are shown in Figure 9. The rPPPE was characterized by one-step degradation with a decomposition onset temperature (T_onset_) of 355 °C, decomposition maximum temperature (T_d max_) of 442 °C and major weight loss (93%) occurring in the 253 to 492 °C temperature range. The fibers degrade in three stages, which corresponds to water loss and subsequent decomposition of the hemicellulose, cellulose and lignin. More detailed analysis on the TGA of the selected fibers has been previously reported [20]. Because of the chemical components of natural fibers, the investigated composites also demonstrated multi-stage decompositions, although they were less pronounced than in the case of the neat fibers. Due to the addition of RS, BS or SCS to the rPPPE, the main decomposition range increased; for example, at 40% concentration of BS in the composite, the DTG peak width expanded by up to 60%. In the case of the composites with lower fiber amounts, the width of the DTG peak changed less (within 20%). Meanwhile, the weight loss of the composites at 600 °C remained at the same level ~93%. At the same time, the composites exhibited a decrease in T_onset_ and T_d max_ with increasing concentration of the fibers in the rPPPE matrix. The T_onset_ values changed within the range of 17%, especially in the case of the BS-containing composite with 40% fibers, demonstrating a T_onset_ that was 60 °C lower than that for rPPPE. Meanwhile, the smallest changes (3%) in T_onset_ were observed for the composites with an RS concentration of 10% (decrease from 355 to 346 °C). The changes in T_d max_ were smaller, up to 8% for the RS and SCS composites and only 4% for the BS composite. Consequently, the T_d max_ of the composites at the highest fiber loading reach 407 °C (RS, SCS) and 423 °C (BS).

Considering that the fibers reduced the thermal stability of the material, the weight loss at the selected processing temperature (190 °C) was also evaluated. From the results it was found that for most compositions, the mass change in this range was below 1%, which could be attributed to humidity. The highest weight loss (2.5%) was observed for the composite with 40% RS which could indicate a small part of the natural fibers decomposed. In general, the investigated composites can be produced by a melt processing up to 190 °C without considerable structure destruction.

## 4. Conclusions

The investigated composites based on rPPPE showed considerable changes in rheological, mechanical and moisture absorption properties by increasing the concentration of different types of lignocellulosic fibers from SCS, RS and BS biomass residues. The complex viscosity, storage and loss modules of the composites increased with addition of the fibers while the LVE region decreased. Composites with a fiber amount of 40% demonstrated the highest tensile and flexural modules but also the lowest ultimate elongation values. The impact resistance of the composites also decreased by increasing the fiber content. The best effect of the reinforcement was observed from the results of the flexural test, especially in the BS composites. After water immersion, the reinforcement effect of the fibers slightly increased for the 10% composites but decreased for the 40% composites. In general, the study shows that rPPPE can be used to replace virgin polymer matrices; in addition, RS, BS and SCS, obtained from agricultural biomass waste, may be used as reinforcement instead of wood flour. However, the interfacial bonding between the matrix polymer and the fibers should be improved by assessing the use of another compatibilizer and/or optimizing the fibers’ treatment procedure, which the focus of our ongoing investigations. Because the addition of RS, BS and SCS fibers to the rPPPE matrix decreased the decomposition onset temperature and maximal thermal decomposition temperature, the processing temperature of the composites should not exceed 190 °C.

## Figures and Tables

**Table 1:** Composition of the developed rPPPE composites with BS, SCS and RS fibers.

Samples	Matrix Content (wt.%)	Fibers Content (wt.%)	MAH (wt.%)	IRGA (wt.%)
rPPPE	100	-	-	-
10% BS	90	10	3	1
20% BS	80	20		
30% BS	70	30		
40% BS	60	40		
10% SCS	90	10		
20% SCS	80	20		
30% SCS	70	30		
40% SCS	60	40		
10% RS	90	10		
20% RS	80	20		
30% RS	70	30		
40% RS	60	40		

**Table 2:** The crossover points Gc (G′ = G″) at 190 °C of the investigated composites.

Fiber wt.%	Gc(Pa)_BS	Gc(Pa)_SCS	Gc(Pa)_RS
0	23,695	23,695	23,695
10	22,666	39,604	27,716
20	33,168	48,479	28,062
30	40,269	75,043	40,225
40	63,921	89,540	50,044

## Data Availability

Not applicable.

## Supplementary Materials

The following supporting information can be downloaded at: https://www.mdpi.com/article/10.3390/polym15122672/s1, Table S1: Tensile modulus (E_T_), tensile strength (σ_Y_) and strain at break (ε_B_) of rPPPE composites with BS, SCS, and RS fibers. Table S2: Flexural modulus (E_F_), maximum flexural stress (σ_max_) and strain at maximum flexural strength (ε_max_) of rPPPE composites with BS, SCS, and RS fibers before and after immersion in water.
