# Supplementary material for: Effect of Agricultural Biomass Residues on the Properties of Recycled Polypropylene/Polyethylene Composites
Source: Polymers (Basel). 2023 Jun 14;15(12):2672. doi: 10.3390/polym15122672 (PMC10301888; doi:10.3390/polym15122672)
Supplement: Supplementary file 1 [file polymers-15-02672-s001.zip › Table S1.pdf]

Table S1. Tensile modulus (E_T), tensile strength(σ_Y) and strain at break (ε_B) of rPPPE composites with BS, SCS, and RS fibers.

Fiber type	Fiber content, %	E_T, MPa	±STDV	σ_Y, MPa	±STDV	ε_B, %	±STDV
	0	1960	67	39	1.1	59	11.8
BS	10	2269	100	39	1.2	8	1.0
	20	2751	60	40	0.8	7	1.2
	30	3211	80	40	0.6	4	0.6
	40	4359	139	44	0.6	3	0.2
RS	10	2175	112	37	0.4	14	2.7
	20	2799	89	38	0.4	6	1.3
	30	3468	62	38	0.5	3	0.5
	40	4620	106	41	0.1	2	0.8
SCS	10	2205	57	37	0.4	7	1.6
	20	2728	191	43	0.8	6	1.1
	30	3546	67	40	0.5	4	0.5
	40	4060	100	40	0.7	3	0.3
