# Supplementary material for: Effect of Agricultural Biomass Residues on the Properties of Recycled Polypropylene/Polyethylene Composites
Source: Polymers (Basel). 2023 Jun 14;15(12):2672. doi: 10.3390/polym15122672 (PMC10301888; doi:10.3390/polym15122672)
Supplement: Supplementary file 1 [file polymers-15-02672-s001.zip › Table S2.pdf]

Table S2. Flexural modulus (E_F), maximum flexural stress (σ_{max}) and strain at maximum flexural strength (ε_{max}) of rPPPE composites with BS, SCS, and RS fibers before and after immersion in water.

Fiber type	Fiber content, %	E_F, MPa	±STDV	σ_{MAX}, MPa	±STDV	ε_{MAX}, %	±STDV
	0	1427	79	40	1.1	7	0.1
BS	10	1861	36	47	0.4	6	0.3
	20	2378	108	50	0.3	5	0.1
	30	3220	39	57	0.1	4	0.1
	40	3930	60	60	1.0	3	0.2
RS	10	1744	47	43	0.5	6	0.1
	20	2308	102	49	0.8	5	0.2
	30	2724	127	50	1.3	4	0.1
	40	3615	45	55	0.5	3	0.1
SCS	10	1865	100	48	0.5	6	0.2
	20	2125	39	50	0.7	6	0.1
	30	2540	20	49	1.1	4	0.2
	40	2933	78	51	0.8	4	0.1
				**after immersion**			
	0	1677	19	44	0.2	7	0.1
BS	10	2218	4	52	0.4	6	0.1
	40	2860	195	52	0.4	3	0.3
RS	10	2161	49	51	0.6	6	0.0
	40	2900	68	48	0.9	3	0.2
AS	10	2096	12	52	0.2	6	0.0
	40	3026	26	53	0.7	4	0.2
